# A Novel Biodegradable Polycaprolactone Fixator for Osteosynthesis Surgery of Rib Fracture: *In Vitro* and *in Vivo* Study

**DOI:** 10.3390/ma8115415

**Published:** 2015-11-13

**Authors:** Yi-Hsun Yu, Chin-Lung Fan, Yung-Heng Hsu, Ying-Chao Chou, Steve W. N. Ueng, Shih-Jung Liu

**Affiliations:** 1 Department of Orthopedic Surgery, Musculoskeletal Research Center, Chang Gung Memorial Hospital, Tao-Yuan 33305, Taiwan; alanyu1007@gmail.com (Y.-H.Y.); laurencehsu.hsu@gmail.com (Y.-H.H.); enjoycu@ms22.hinet.net (Y.-C.C.); wenneng@adm.cgmh.org.tw (S.W.N.U.); 2 Department of Mechanical Engineering, Chang Gung University, Tao-Yuan 33302, Taiwan; willy79814@gmail.com

**Keywords:** polycaprolactone, rib fracture, osteosynthesis, internal fixation

## Abstract

Osteosynthesis surgery for rib fractures is controversial and challenging. This study developed a noval poly(ε-caprolactone) (PCL)-based biodegradable “cable-tie” fixator for osteosynthesis surgery for rib fractures. A biodegradable fixator specifically for fractured ribs was designed and fabricated by a micro-injection molding machine in our laboratory. The fixator has three belts that could be passed through matching holes individually. The locking mechanism allows the belt movement to move in only one direction. To examine the *in vitro* biomechanical performance, ribs 3–7 from four fresh New Zealand rabbits were employed. The load to failure and stress-strain curve was compared in the three-point bending test among native ribs, titanium plate-fixed ribs, and PCL fixator-fixed ribs. In the *in vivo* animal study, the sixth ribs of New Zealand rabbits were osteotomized and osteosynthesis surgery was performed using the PCL fixator. Outcomes were assessed by monthly X-ray examinations, a final micro-computed tomography (CT) scan, and histological analysis. The experimental results suggested that the ribs fixed with the PCL fixator were significantly less stiff than those fixed with titanium plates (*p* < 0.05). All ribs fixed with the PCL fixators exhibited union. The bridging callus was confirmed by gross, radiographic micro-three-dimensional (3D) CT, and histological examinations. In addition, there was no significant inflammatory response of the osteotomized ribs or the PCL-rib interface during application. The novel PCL fixator developed in this work achieves satisfactory results in osteosynthesis surgery for rib fractures, and may provide potential applications in other orthopedic surgeries.

## 1. Introduction

Rib fractures are not uncommon injuries in trauma care hospitals and are usually caused by blunt chest trauma [1,2,3]. Rib fracture can be a solitary injury or merely one injury in a polytrauma patient. Nonsurgical treatment has been traditionally favored not only because the fracture can heal completely without surgery, but also because of the lack of risks and complications of surgery, such as iatrogenic pulmonary injury and loosening of the metal implants intra- or post operatively. However, during the bone healing process, severe pain as a result of breathing may cause poor pulmonary hygiene and lead to complications such as atelectasis and pneumonia, consequently prolonging the period of intubation and mechanical ventilation [1,4].

At present, internal fixation for multiple rib fractures has the advantages of substantially diminishing pain and discomfort, shortening the duration of mechanical ventilation, and preventing malunion and nonunion [4]. The recommended indications for osteosynthesis surgery include special conditions such as multiple rib fractures, flail chest, acute intractable pain, open chest wall defect, pulmonary herniation, and painful nonunion [5]. Implants for the internal fixation of a fractured rib can be metallic or biodegradable. Commercially available metallic rib implants comprise a plate, clamp, and intramedullary wire. The most obvious advantage of these implants is their excellent mechanical strength and stability. However, their disadvantages include the “stress-shielding effect” as well as their high frequency of loosening due to the dynamic movement of the thoracic cage during breathing. Finally, once the fractured bone heals, metallic implants must always be removed because of their prominence [6].

On the other hand, biodegradable implants can provide adequate mechanical strength for bone union but do not need to be removed, as they degrade within the body. Biodegradable implants for the ribs are mainly made of polyglycolic acid (PGA) or polylactide acid (PLA). However, degradation times inadequate for bone union might cause early failure before the fracture is united. Furthermore, although previous studies have evaluated biodegradable plates for rib fixation, the plates were not designed specifically for rib fractures [1,7,8].

We assume that a biodegradable fixator should provide the needed mechanical support for rib fixation while removing the need for a secondary operation to move the fixator. In this study, a novel biodegradable “cable-tie”-type fixator made of biodegradable poly(ε-caprolactone) was developed and fabricated using a micro-injection molding technique. Polycaprolactone (PCL) is a semi-crystalline polyester with a low melting point of approximately 60 °C and a glass transition temperature of about −60 °C [9]. It is degraded by hydrolysis of its ester linkages in physiological conditions and has been one of the most promising biodegradable biomaterials. PCL is non-toxic and tissue-compatible, and can be eventually be resorbed in the vital organs. In particular PCL is especially interesting for the preparation of long-term implantable devices due to its degradation which is even slower than that of polylactide. To manufacture the biodegradable fixator, a lab-scale micro-injection molding machine was employed. After molding, the effectiveness of the fixator for the surgical fixation of rib fractures was investigated. In the biomechanical study, we tested the mechanical strength of the PCL fixator over fractured rabbit ribs and compared it to a titanium miniplate. Finally, the PCL fixator was applied to the rabbit rib fracture model to evaluate its ability and effectiveness for osteosynthesis.

## 2. Materials and Methods

### 2.1. Fabrication of the Biodegradable PCL Fixator

**Figure 1:**
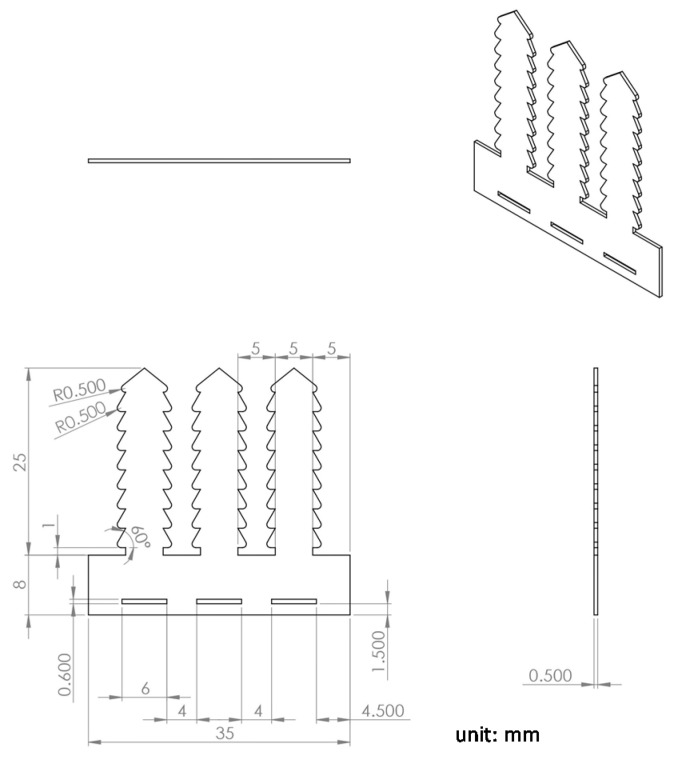
Layout and dimensions of the polycaprolactone (PCL) fixator.

A biodegradable fixator specifically for fractured ribs was designed and fabricated in our laboratory. The implant was made of poly(ε-caprolactone) (PCL), with an average molecular weight of 80,000 Da (Sigma-Aldrich Co. LLC., St. Louis, MO, USA). The fixator was fabricated by a micro-injection molding machine (Fanuc Roboshot S-2000i 15A, Fanuc, Oshino-mura, Japan) in our laboratory according to our previously described procedure [10]. Three belts that could be passed through matching holes individually were designed. The locking mechanism allows the belt movement to move in only one direction (Figure 1); it secures the rib to the fixator so that no screw-plate fixation mode is required to fix the fractured rib.

### 2.2. In Vitro Biomechanical Study

Before the *in vivo* animal tests, a preliminary *in vitro* study was first carried out to examine the mechanical property of the developed PCL fixator. Four six-month-old New Zealand white rabbits weighing 2800–3200 grams were used in the biomechanical study. Carbon dioxide was used for euthanasia. The rabbit was placed in the chamber and then 100% carbon dioxide was introduced. A fill rate of 2 L/min of carbon dioxide in the chamber was performed and lasted for 3 min. Carbon dioxide was maintained for 2 min after the observation of the lack of respiration and faded eye color. Then the rabbit was sacrificed. All animal procedures received institutional approval from the Institutional Animal Care and Use Committee (IACUC) at the Animal Center of Chang Gung University (CGU 14-102). All studied animals were cared for in accordance with regulations of the National Institutes of Health of Taiwan.

The third through seventh unilateral ribs were harvested. A total of 20 ribs were examined. The ribs were excised, and the surrounding soft tissue and muscle were cleared. The entire experiment was performed in our laboratory under controlled temperature (25–27 °C). The biomechanical experiment was a three-point bending test performed on a mechanical testing device (LRX, Lloyd Instruments, Bognor Regis, UK). A native rib was placed on the supporting bars 25 mm apart, which were moved at 1 mm/s to create fractures at the end of each test. The applied force and strain throughout the force accumulation were recorded. The stress–strain curve was also created for each tested rib.

**Figure 2:**
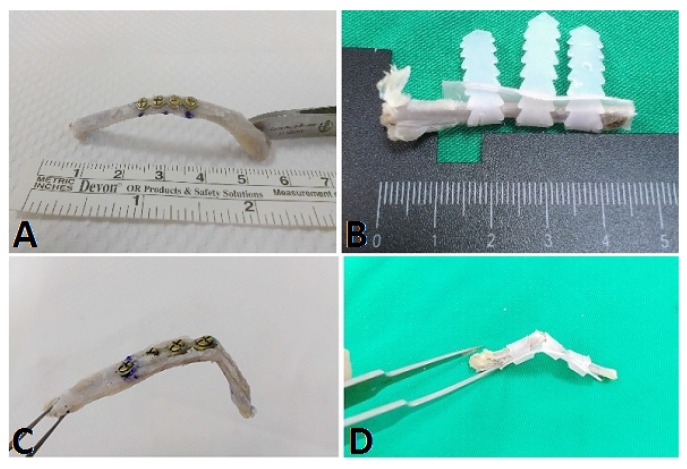
The rib fracture model fixed by (**A**) titanium miniplate and (**B**) PCL fixator. During the three-point bending biomechanical study, (**C**) a new fracture was observed at the edge of the miniplate in the Ti group and (**D**) the locked mechanism remained intact in the PCL fixator group.

The tested specimens were then randomly divided into two groups to be fixed either by a titanium miniplate (Ti group) (Synthes, Paoli, PA, USA) or the PCL fixator (PCL group). In the Ti group, a four-hole, 0.5 mm thick, and 15 mm long titanium plate was used, with two holes at each end of the fracture site (Figure 2A). The matching titanium screws had a core diameter of 1.3 mm. Meanwhile, in the PCL group, one belt at each end of the fracture site was attached and another was just passed around the fracture site. All three belts were pulled and tightened after entering the matching holes (Figure 2B). After fixation, the implant-rib composites were reexamined under the same parameters as the three-point bending test.

### 2.3. Animal Model

**Figure 3:**
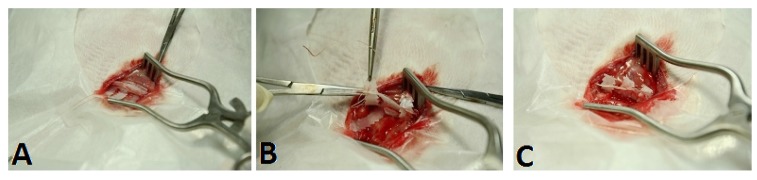
Surgical procedure of the PCL cable-tie fixator. (**A**) Passed the belt beneath the rib; (**B**) Locked the belt; (**C**) Final fixation.

Six six-month-old New Zealand white rabbits weighing 2800–3200 grams were used in the animal model. The sixth rib of the right chest wall was selected as the target rib for the fracture model. Before the surgical procedure, the animal was restrained manually and oxygen was delivered by facemask for 5 min at 4 L/min. Following pre-oxygenation, isoflurane inhalation was delivered by the facemask until the animal was anesthetized. Isoflurane inhalation was maintained throughout the surgical procedure. The rabbit was placed in the decubitus position, with the surgical field upward. The skin was prepared and disinfected according to standard procedures. A 2.5 cm skin incision was made, directly above the target rib, and the soft tissue and muscular layer of the rib were dissected carefully. The osteotomy was created in a short oblique direction. Then, the fractured rib was fixed by the PCL fixator (Figure 3). If the pleura was injured and potential pneumothorax was suspected, a prophylactic or therapeutic chest tube was inserted. The wound was subsequently closed in layers with 2-0 and 3-0 Dexon sutures. Post-operatively, the rabbits were observed for post-anesthesia condition and returned to their cages when they were alert and could walk. The rabbits were cared for in accordance with the institutional guidelines of Chang Gung University.

The rabbits underwent X-ray examination each month post-operatively. Before the X-ray examination, the rabbits were sedated with an intravenous zolazepam and tiletamine injection (Zoletil, Virbac Taiwan Co., Ltd., Taipei, Taiwan). X-rays were taken in anteroposterior and oblique views. The rabbits were sacrificed according to standard euthanasia procedure three months post-operatively. The target rib was excised, and the surrounding soft tissue was cleaned and sent for histological examination to investigate the inflammatory response after PCL implantation. After releasing the locking mechanism of the fixator, the rib was examined grossly and sent for micro-CT and histological examinations.

### 2.4. Statistical Analysis

The data were analyzed by using commercial SPSS software version 18.0 (INC., SPSS, Chicago, IL, USA). Continuous variables are presented as means and standard deviations. The variables were compared among the control, Ti and PCL groups by using parametric *t*-tests for independent parameters and paired *t*-test for related parameters. A two-tailed *p*-value less than 0.05 was considered significant.

## 3. Results

### 3.1. Biomechanical Study

**Figure 4:**
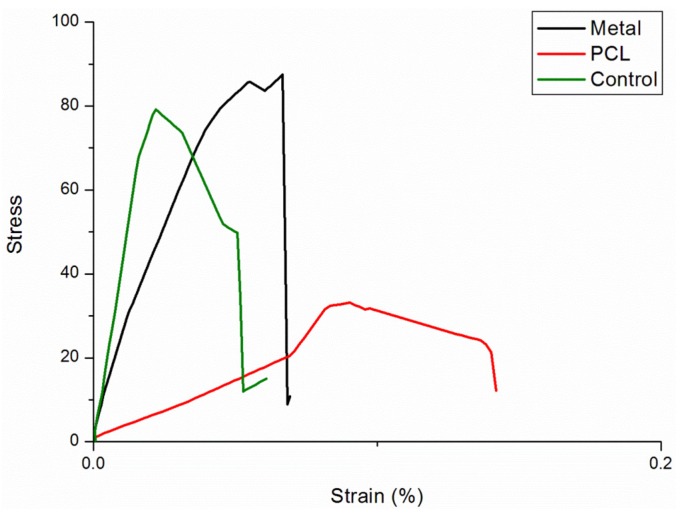
The stress-strain curves of the native ribs (green), Ti-group (black), and PCL group (red). The ribs in the Ti group can support the highest stress; the ribs in the PCL group can support the lowest stress with the highest deformity.

A total of 20 ribs from four rabbits were tested. The result of the three-point bending test of the native ribs, Ti group, and PCL group are shown in Table 1. There was no significant difference in the force required to cause failure between the native ribs and the Ti group. In the PCL group, the force required to cause failure was significantly lower than that in the native and Ti groups. The stress-strain curves are shown in Figure 4.

**Table 1:** The result of the three-point bending test of the native ribs, Ti group, and polycaprolactone (PCL) group.

**Native Rib Parameters**			
Experimental results	Native ribs		
	Ti group (*n* = 10)	PCL group (*n* = 10)	*p*-value
Mean load at failure (N)	16.27 ± 4.13	18.59 ± 4.74	0.26
Mean stress at failure (MPa)	58.46 ± 8.33	64.2 ± 16.50	0.50
Mean strain (mm)	0.03 ± 0.01	0.03 ± 0.01	0.11
**Comparison between Native Ribs and Ribs in Ti Group**			
Experimental results	Native ribs	Ti group	*p*-value
Mean load at failure (N)	16.27 ± 4.13	17.12 ± 3.45	0.44
Mean stress at failure (MPa)	58.46 ± 8.33	70.82 ± 35.40	0.14
Mean strain (mm)	0.03 ± 0.01	0.12 ± 0.22	0.25
**Comparison between Native Ribs and Ribs in PCL Group**			
Experimental results	Native ribs	PCL group	*p*-value
Mean load at failure (N)	18.59 ± 4.74	4.64 ± 2.75	0.005
Mean stress at failure (MPa)	64.2 ± 16.50	14.78 ± 13.16	0.005
Mean strain (mm)	0.03 ± 0.01	0.11 ± 0.03	0.005
**Comparison between Ribs in Ti Group and in PCL Group**			
Experimental results	Ti group	PCL group	*p*-value
Mean load at failure (N)	17.12 ± 3.45	4,64 ± 2.75	<0.001
Mean stress at failure (MPa)	70.82 ± 35.40	14.78 ± 13.16	<0.001
Mean strain (mm)	0.12 ± 0.22	0.11 ± 0.03	0.005

During the three-point bending test, there was no loosening of the screws in the Ti group. New fractures were created in all ribs in the Ti group as the loading force exceeded the maximal loading force. The new fracture was located just at the junction of the edge of the titanium plate and the rib (Figure 2C). In the PCL group, all locking mechanisms of the PCL fixators remained intact, no breakage of the PCL fixators occurred, and no new fractures were created as the loading force increased. The ribs were completely secured by the PCL fixator (Figure 2D).

### 3.2. Animal Study

**Figure 5:**
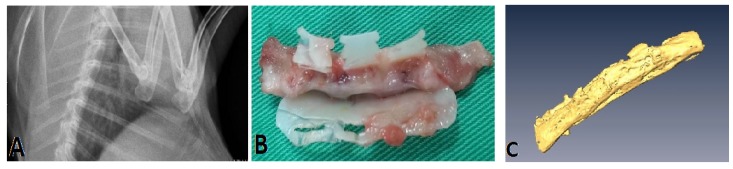
Results after the osteosynthesis surgery for the rib osteomized by PCL fixator. (**A**) The osteomized rib showed union on the 12-week follow-up X-ray; (**B**) The specimen showed the osteomized rib with good callus formation grossly; (**C**) Three-dimensional CT reconstruction showed good rib cortex continuity and callus formation.

Six osteotomized ribs from the six New Zealand rabbits were fixed with the PCL fixators. Monthly follow-up X-rays showed the fracture sites were secured and there was no displacement of the osteotomized ribs under fixation. All rabbits survived the surgery; they were sacrificed 12 weeks post-operatively, and the specimens were obtained. All fractured ribs were united grossly (Figure 5A,B). Three-dimensional micro-CT showed excellent bone continuity and callus formation (Figure 5C).

### 3.3. Histological Examination

**Figure 6:**
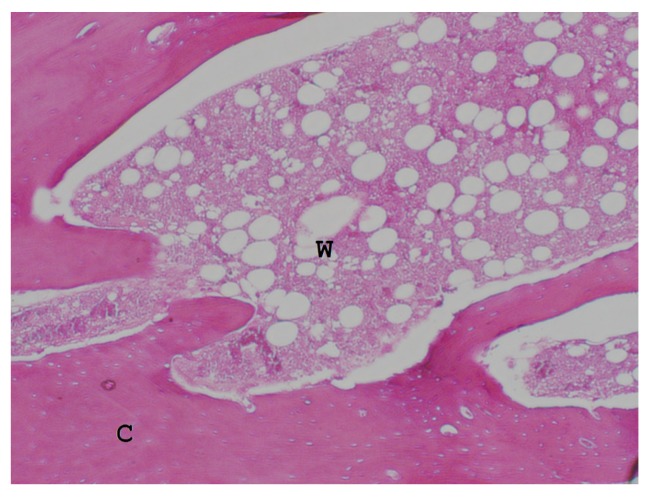
The histology of the callus showed a mixture of cortical bone (C) and woven bone (W).

Histological examination of the decalcified specimens showed the remolding callus comprised cortical and woven bone (Figure 6). There were no foreign body giant cells or inflammatory cells around the osteotomized rib, the PCL-rib interface, or the surrounding soft tissue.

## 4. Discussion

There are currently five biodegradable materials approved for medical uses by the US Food and Drug Administration. Among these materials, PCL has been used in medicine since the 1930s [9]. PCL, a biocompatible and biodegradable aliphatic polyester, is now mostly used in sutures, wound dressings, drug delivery devices, and tissue engineering applications. In bone engineering in particular, PCL is used to enhance bone ingrowth and regeneration in the treatment of bone defects [11,12].

In orthopedic surgeries, most commercially available biodegradable implants are made of either homopolymers (e.g., PGA, PLA and polydioxanone) or copolymers of PGA and PLA [13]. In this study, we selected PCL as the material for the novel fracture fixator for two reasons. First, fracture healing takes time. Although fracture healing usually takes 12 weeks in rabbit models, it usually takes 9–12 months for complete callus consolidation in humans. Both PGA and PLA biodegradable bone implants only retain structural integrity and strength for less than 12 weeks before consequently undergoing hydrolysis and degradation [13,14]. In contrast, PCL only begins to degrade at least 24 months after implantation *in vivo* [15,16], and its mechanical strength can be maintained for up to 33 weeks [17]. Therefore, PCL’s long degradation time is suitable for fracture healing applications in humans. Second, acid-base changes regulate bone metabolism. An acidic environment may upregulate osteoclasts and downregulate osteoblasts [18,19,20,21]. The degradation products of PGA and PLA yield lactic acid at the callus formation stage of the bone healing process; therefore, the acidic environment may retard the normal bone healing process [22,23]. Although PCL degradation products also contribute to an acidic environment, the fracture healing process may be completed before PCL begins to degrade. Furthermore, PCL is in its semi-crystalline state at room temperature and thus exhibits a good flexibility. This would provide advantages in terms of deployment of the fixator to the fractured ribs.

For fracture fixation, the notion that the strongest fixation strength results in the best clinical outcomes does not always hold true. The gold standard for the treatment of femoral shaft fracture is fixation with intramedullary nailing, as this method allows some micromotion of the fracture gap, consequently stimulating callus formation and inducing the secondary bone healing pathway. The same basis can be applied to rib fracture fixation. The thoracic cage undergoes dynamic motion during breathing. The strong fixation of metallic plates may be loosened because of periodic thoracic cage movement. Other disadvantages of metallic plates include the stress-shielding effect, delayed union, and bone atrophy. Biodegradable bone implants allow stress loads to gradually be returned to the bone as the implant is degraded; thus, the bone bears more of the load as the fracture heals, preventing stress shielding [24]. In addition, the PCL cable-tie fixation allowed some micromotion across the fracture site during breathing, which also assists healing.

Despite the fact that satisfactory results were obtained in the present experiment, this study has some limitations that should be mentioned. First, we created a wound large enough to embed the PCL fixator. The current trend in orthopedic practice is minimally invasive osteosynthesis surgery due to its advantages. Our PCL fixator is merely a preliminary design, so the wound had to be sufficiently large. Further designs allowing the biodegradable PCL fixator to be placed with less invasive surgery will be developed for better clinical application and outcomes. Second, the observation period of the animal study only lasted three months, by which time PCL had not degraded. We observed minimal inflammatory response around the PCL implant grossly and microscopically. Future investigations should compare PCL implants and existing titanium implants for longer implantation durations, as well as evaluate the degradation behavior of PCL materials.

## 5. Conclusions

This study has developed a novel biodegradable PCL fixator that represents a new and innovative design for the fixation of rib fractures. Biomechanical and animal studies demonstrated satisfactory results with this fixator. No significant inflammatory response of the osteotomized ribs or the PCL-rib interface was observed during application. Further generations of the PCL fixator should be developed in order to facilitate less invasive surgery and application. Eventually the novel PCL fixator developed in this work may be used in humans for the treatment of rib fractures as well as in other orthopedic surgeries.
